# Functionalized Noble Metal Nanoparticles for the Treatment of Herpesvirus Infection

**DOI:** 10.3390/microorganisms10112161

**Published:** 2022-10-31

**Authors:** Martyna Janicka, Katarzyna Ranoszek-Soliwoda, Grzegorz Chodaczek, Małgorzata Antos-Bielska, Marek Brytan, Emilia Tomaszewska, Grzegorz Celichowski, Jarosław Grobelny, Joanna Cymerys, Małgorzata Krzyżowska, Marcin Chodkowski

**Affiliations:** 1 Military Institute of Hygiene and Epidemiology, Kozielska 4, 01-163 Warsaw, Poland; 2 Division of Microbiology, Department of Preclinical Sciences, Institute of Veterinary Medicine, Warsaw University of Life Sciences, 02-786 Warsaw, Poland; 3 Department of Materials Technology and Chemistry, Faculty of Chemistry, University of Lodz, Pomorska 163 St., 90-236 Lodz, Poland; 4 Bioimaging Laboratory, Łukasiewicz Research Network—PORT Polish Center for Technology Development, 54-066 Wroclaw, Poland

**Keywords:** noble metal nanoparticles, HHV-1, neuroinfection

## Abstract

Neuroinfections caused by herpesviruses, mainly by HHV-1, represent a significant problem for modern medicine due to the small number of therapeutic substances available in the pharmaceutical sector. Furthermore, HHV-1 infection has been linked to neurodegenerative processes such as Alzheimer’s disease, which justifies the search for new effective therapies. The development of nanotechnology opens up new possibilities for the treatment of neuroinflammation. Gold and silver nanoparticles are gaining popularity, and the number of clinical trials involving metallic nanoparticles is constantly increasing. This paper reviews the research on gold and silver nanoparticles and their potential use in the treatment of herpesvirus neuroinfection.

## 1. Introduction

Infections of the central nervous system (CNS) are sporadic; they mainly affect pediatric patients, as well as individuals with a compromised immune system, due to immunosuppression or congenital dysfunction of the immune system [1,2,3]. CNS infection can occur in different forms, depending on the biology of the virus; it may take the form of an acute, chronic, or latent infection. Both DNA and RNA viruses can cause neurological diseases, including meningitis, encephalitis, meningovasculitis, radiculitis myelitis, cranial neuritis, and neuritis, potentially involving the entire range of neurological and anatomical sites. So far, many neurotropic viruses have been recognized to cause a range of CNS diseases, but herpesviruses are leading in neuropathogenic infections due to their ability to establish latency in the neuronal tissue (neuronal ganglia) or the lymphatic tissue (B cells) [4,5].

During primary infection, the Herpesviridae family of viruses causes minor dysfunction, including fever and skin or mucous membrane rash [6]. Primary infections are followed by the establishment of the latency state, in which virus particles are not recognized by the immune system, thus giving the virus an “advantage” over the host cell’s defense mechanisms [5]. The mechanisms of herpesviruses’ penetration into the CNS are not fully understood. There are some assumptions that these viruses can spread along with the ganglia or enter the frontal and temporal lobe of the brain directly from the mucous membranes of the upper respiratory tract [4,7].

Human herpesvirus type 1 (HHV-1) is a double-stranded DNA virus classified as an alpha-herpesvirus. HHV-1 can cycle productively and establish a latency state within the CNS. Transmission occurs mostly by direct contact; less commonly, the infection can occur through blood transfusion or transplants. Children usually become infected by their parents during infancy. The disease begins with the interaction of viral glycoproteins with host membrane receptors. The primary sites of HHV-1 infection are mainly oral epithelium, conjunctiva, or genital mucosa. The productive cycle of the virus manifests with vesicular lesions around the mouth and nose. An elevated temperature may accompany symptoms [8,9]. In addition, as mentioned above, the virus establishes latency in the trigeminal ganglia after the productive cycle. During the latency period, only specific viral genes are detected, such as LATs (latency-associated transcripts). Immunosuppression, physical stress, and UV radiation are only a few factors that can reactivate the herpesvirus infection [5]. Many studies indicate that HHV-1 infection of the brain causes neurological complications such as headache, fever, lethargy, confusion, irritability, aphasia, seizures, and focal deficits [10,11]. Herpes simplex encephalitis (HSE) is caused by HHV-1 or, less frequently, type 2 (HHV-2) [5,10,11]. Most commonly, it affects pediatric, immunosuppressed, and HIV-infected patients [11,12]. HSE is caused by the activation of microglia cells, which, upon activation, secrete inflammatory mediators as a response to HHV-1 infections [13]. However, prolonged activation of microglia influences the persistence of HSE, as well as the appearance of its sequelae, including the development of neurodegenerative processes [13,14,15].

In addition, herpesvirus infection has been linked to neurodegenerative diseases such as Alzheimer’s disease. HHV-1 infection affects nerve cell functions [15]. Studies indicate that infection induces the activation of a calcium ion-dependent signaling pathway that induces phosphorylation of the amyloid precursor protein (APP). Consequently, this leads to extracellular and intracellular deposition of APP breakdown products, including amyloid β [16]. Moreover, an increase in autophagy-specific markers has also been reported in HHV-1-infected neurons. Tau protein hyperphosphorylation is also linked with the neurodegeneration processes associated with HHV-1 infection [17,18,19].

## 2. Standard Antiviral Treatment

Since the discovery of nucleoside analogs, treatment strategies for herpesvirus infection have remained unchanged. Antiviral drugs for HHV-1 infection include acyclovir (ACV), famciclovir, and valacyclovir, consisting analogs of viral thymidine kinase phosphorylates nucleotide incorporated into viral genetic material [20]. Acyclovir has the greatest in vitro activity against HHV-1 and HHV-2 and is the most popular antiviral drug. However, famciclovir and valacyclovir have greater oral bioavailability than acyclovir [21]. The widespread use of nucleoside analogs, mainly acyclovir, is associated with increasing drug resistance of HHV-1. In addition to acyclovir, dexamethasone is used to treat herpesviral encephalitis in case of extensive inflammatory changes in the brain [22]. Therefore, it is necessary to search for new therapies that inhibit the productive cycle and prevent the reactivation of the infection. In the literature, there are many data on natural treatments for HHV-1 infection using polyphenols isolated from plants [23]. El-Toumy et al. examined the in vitro antiviral activity of plant extracts derived from Egyptian plants. They found that eight plant extracts and seven pure phenolic compounds out of 25 plant extracts showed strong anti-HHV-1 activity [24]. Among these, the phenolic compounds gallic acid and curcumin showed the strongest anti-HHV-1 activity. Another study demonstrated the ability of four plants traditionally used by women of the Tacana tribe in the Bolivian Amazon to block HHV-2 infection in vitro and in vivo. The plant extracts blocked HHV-2 binding and entry but could not block viral replication after entry. No clinical signs were observed in mice infected with HHV-2 and treated with these extracts, and no virus replication in the reproductive organs and spinal cord was detected [25]. Polyphenols with antiviral activity have also been used for the chemical modification of nanoparticles (NPs) [22,23]. Recently, there has been a lot of discussion about NPs synthesized using photosynthesizing organisms, so-called ‘green synthesis’. Algae, e.g., cyanobacteria, are mainly employed to synthesize such NPs. In the study by Mostafa M. El-Sheekh et al., two species of cyanobacteria were used: *Oscillatoria* sp. and *Spirulina platensis*, to synthesize gold (AuNPs) and silver (AgNPs) nanoparticles. The virucidal potential of obtained compounds was tested against HHV-1 in a Vero cell line. AuNPs and AgNPs produced by the green synthesis showed a reduction in the cytopathic effect induced by HHV-1 [26].

## 3. Noble Metal Nanoparticles as Antiviral Drugs

**Figure 1:**
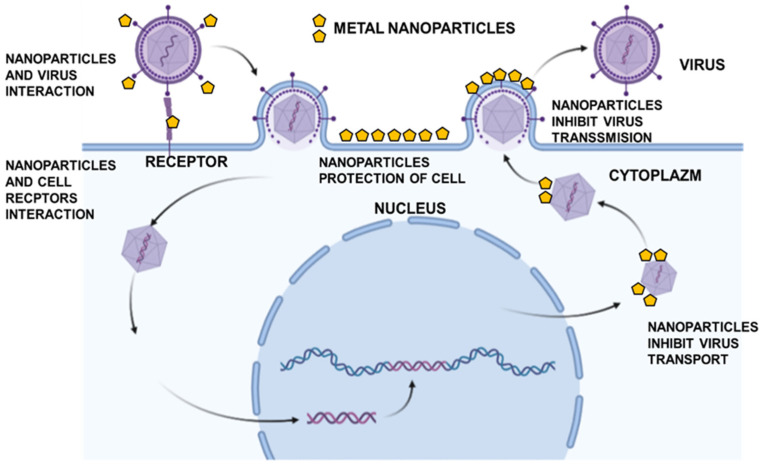
Potential antiviral mechanisms by metal nanoparticles: interaction with cell receptors, interaction with viral particles, inhibition of viral cell-to-cell spreading, and inhibition of virus transport. Based on our own studies, created in BioRender.

Nanotechnology uses particles at the nanoscale of 10^−9^ m. NPs of noble metals have unique chemical, physical, and biological properties mainly due to their small size. Therefore, they can be used as carriers of medicines and can undergo various chemical modifications [27]. The use of gold NPs, which are chemically inert and biologically compatible, seems particularly useful. In recent years, nanotechnologies have been widely researched and developed for antiviral treatment. Compared to traditional antiviral therapies, those at the nanoscale offer many new opportunities [27,28,29]. Nanomaterials can help improve current treatments and can also be used to create new strategies to inhibit viral replication. So far, several strategies for the antiviral action of NPs have been proposed [30]. These include the interaction of NPs with virion, inhibition of viral transport in the cell, inhibition of attachment by blocking the cellular receptor, and interaction of NPs with virus components (Figure 1).

However, much is still unknown about how NPs can inhibit viral replication. Some studies have also shown that the activity of metallic NPs is associated with the stimulation of the immune system and antiviral immunity. The mode of penetration and toxicity of metallic NPs are the main limitations of their therapeutic use. The NP penetration depends on their size and chemical modification; NPs penetrate cells by phagocytosis, micropinocytosis, caoevolin-dependent, and clathrin-dependent endocytosis or directly pass through the cellular membranes [31]. Cytotoxicity depends strongly on the NP size, modification, and sensitivity of the particular cell type of the tested metal. Thus, cytotoxicity assays should precede the testing of virucidal activity [32]. Furthermore, both antiviral and immune stimulatory activity may be related to the ability of modified nanoparticles to interact either with the virion’s surface or with the specific viral receptor present on the cell surface (Table 1). Herpesviruses use heparan sulfate proteoglycans (HSPGs) to initiate binding with the target cell. The binding between herpesviruses and HSPGs occurs mostly between closely packed basic amino acids present in viral surface proteins and called viral attachment ligands (VALs). For HHV-1 and HHV-2, the VALs consist of gB and gC viral glycoproteins [5]. Therefore, modified nanoparticles can bind either to HSPGs or VALs and block virus attachment and penetration into the host cell [28,29].

For the treatment of neuroinfections, the manner by which NPs enter the CNS seems to be crucial. Both Ag and AuNPs can overcome the blood–brain barrier (BBB). The penetration of NPs into the brain depends on their size and surface modifications. Most often, NPs overcome the barrier with the involvement of immune cells and cause local barrier disruption. The BBB is essential for the homeostasis of the CNS and serves as a link between the brain and the body. It is responsible for the entry of nutrients, ions, and vitamins. The BBB is made up of brain microvascular endothelial cells, tight junctions, astrocytes, pericytes, and the basal membrane [38]. An important role in the continuity of the BBB is played by tight junctions. A number of studies indicate that claudin 5 and occludin significantly influence the maintenance of a normal BBB. A study by He et al. showed that there is a significant decrease in claudin 5 and ccluding protein levels during HSE [39]. Due to the complex structure of the BBB and selective permeability, the treatment of neurodegenerative diseases such as Alzheimer’s or Parkinson’s disease and neuroinfections by HHV-1 is difficult. Most of the proposed substances with therapeutic potential have little ability to overcome the BBB. Only small molecules, such as water, some gases, and lipid-soluble compounds, can easily cross the BBB by simple diffusion. The transport of large molecules with a high electrical charge, polarity, and hydrophilicity, such as glucose, amino acids, and most drugs, is active and requires the involvement of specialized proteins [40].

## 4. Gold Nanoparticles

**Figure 2:**
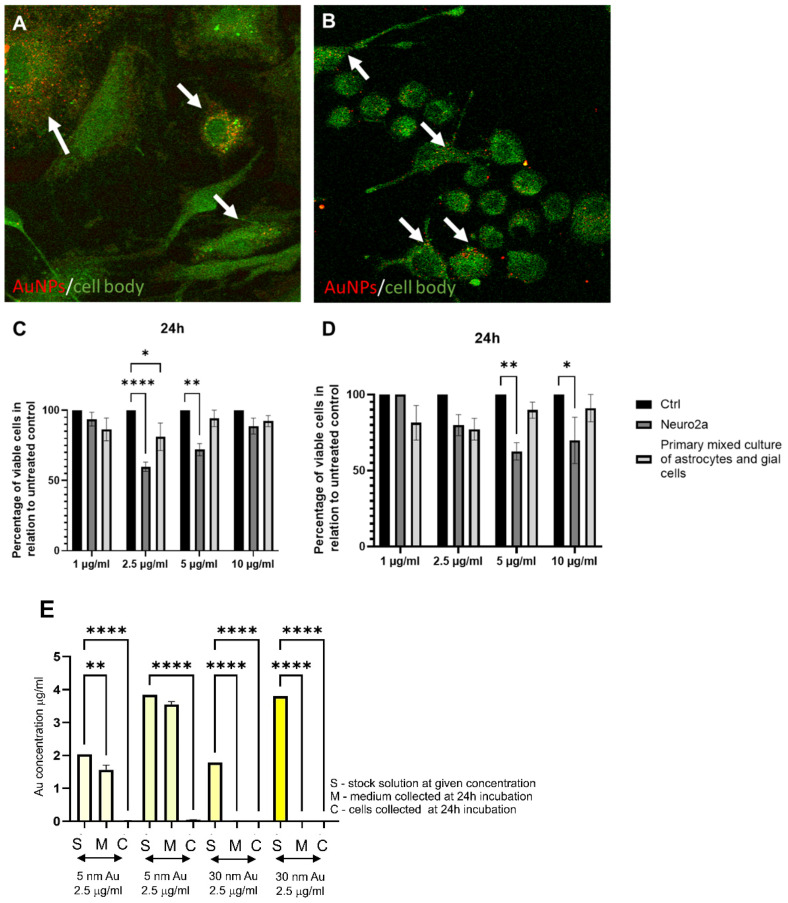
AuNPs internalization by confocal microscopy, ICP-MS and size/cell type-dependent toxicity. Primary mixed culture of astrocytes and glial cells (**A**), forskolin differentiated Neuro-2a cells (**B**) were treated with tannic acid modified AuNPs (TA-AuNPs) 30 nm at a concentration of 2.5 µg/mL for 24 h. For microscopic detection of gold NPs, cells were plated on slides for 18 h before exposure to NPs. After 24 h of exposure to TA-AuNPs, the medium was discarded, and the cells were fixed with acetone:methanol; 1:1 (Sigma-Aldrich, St. Luis, MO, USA). The images were captured on an upright Leica SP8 resonant scanning confocal system (Leica Microsystem, Wetzlar, Germany). The pinhole was set to 1 AU. Nanoparticles were visualized in a reflection mode (red color) using a 638 nm laser line with a 40× oil immersion objective (NA 1.30), while cellular autofluorescence (green color) was used to visualize the cell shape. Arrows point to nanoparticles (red). The acquisition was performed in a sequential mode. The 5 nm (**C**) and 30 nm TA-AuNPs (**D**) cytotoxicity was estimated by the MTT test. The cell viability was measured in Neuro-2a and primary mixed culture of astrocytes and glial cells incubated with various concentrations of TA-AuNPs for 24 h. Control is an untreated cell culture, acting as 100% viability. (**E**) Inductively coupled plasma mass spectrometry (ICP-MS) for Au content in stock solutions, media, and cells collected at 24 h post incubation. Data from three independent experiments are presented as mean ± SEM. Two-way ANOVA test *p* ≤ 0.05 *, *p* ≤ 0.01 **, and extremally significant at *p* ≤ 0.0001 ****. The figure presents our own unpublished data.

**Figure 3:**
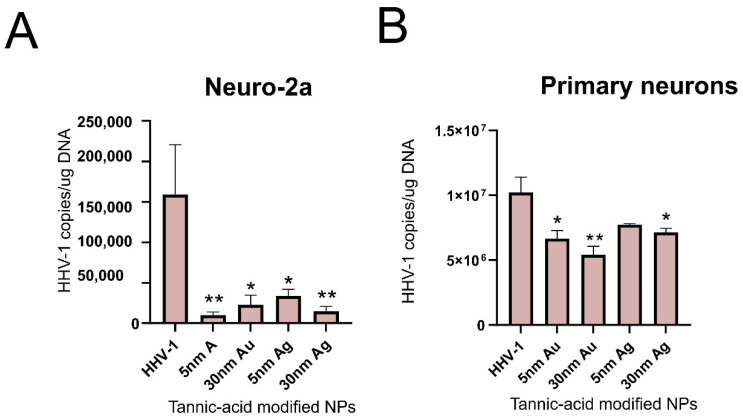
Tannic-acid modified Ag/AuNPs (TA- Ag/AuNPs) block HHV-1 infection in Neuro-2a cell line (**A**) and primary neurons culture (**B**). Both cell cultures were pre-treated for 6 h with a non-toxic concentration of tannic acid-modified AgNPs, or AuNPs sized 5 and 30 nm (2.5 µg/mL). At 24 h p.i., cells were subjected to HHV-1 copies titration by qPCR. The data are expressed as means from three independent experiments ± SEM. * represents significant differences with *p* ≤ 0.05, ** *p* ≤ 0.01 in comparison to untreated infected control (two-way ANOVA test). The figure presents our own unpublished data.

Gold nanoparticles (AuNPs) are widely used in medicine and pharmacy due to their excellent biocompatibility, related to their chemical stability and physical properties. Due to their intrinsic properties, AuNPs are easily chemically modified by the attachment of compounds such as proteins, enzymes, or chemotherapeutic agents. Chemical modification of AuNPs allows for the formation of cell- and molecular-specific NPs. They can be developed to bind specifically to voltage-gated sodium channels, transient receptor potential vanilloid member 1 (TRPV1) channels, and P2X_3_ receptor ion channels in dorsal root ganglion neurons via antibodies as the AuNP-anchoring molecules [41]. More importantly, AuNPs have also been shown to act as a photothermal agent, heating only the area of interaction with NPs, serving to alter neuronal activity [42]. AuNPs are increasingly used in neuroscience due to their various properties, such as stimulating neuronal activity or increasing the neurite length (can be used in regenerative medicine). Moreover, they are not toxigenic and provide a good research model to study their effects against neuroinfections caused by HHV-1 (Figure 2). In addition, they have also been used in altering the polarity of nerve cells and in calcium signaling necessary for nerve impulse transmission [43,44]. Furthermore, many studies have shown the neuroprotective effects of gold-cored NPs [45]. Studies by Madhusudanan et al. confirmed that large AuNPs above 15 nm influence cell tolerance and can stimulate an increase in neurite length. In addition, other studies indicate an increased number of neurites in cell cultures treated with AuNPs [46]. Furthermore, 30 nm AuNPs are internalized by the cells showing microglial morphology (Figure 3A) but very modestly by neuronal cells (Figure 3B,E), as indicated by our in vitro studies. 

AuNPs can be effectively used to treat neuroinfection caused by HHV-1. A study by Rodriguez-Izquierdo et al. showed that AuNPs could inhibit HHV-1 infection in a neural-derived SK-N-MC cell line model. Antiviral activity was related to the number of sulphonate groups coating the NPs. It appeared that the more sulfonate end groups, the greater the inhibition of infection in the treatment (addition of AuNPs after the infection) and pretreatment (addition of AuNPs before the infection) test [45].

AuNPs have been shown to overcome the BBB, and their effects on brain tissue have already been studied. Mice were injected with AuNPs via an intravenous route, and histopathological changes in the brain were assessed by evaluating the presence of inflammatory infiltration, the presence of microglia around neurons, the degree of vacuolization of neuronal cells, and the detection of neovascular changes such as edema. It was found that AuNPs did not induce any brain changes, and inflammatory infiltration was observed in only a few specimens. Moreover, it has also been confirmed that their cytotoxicity is closely related to their size and chemical modification. No histopathological changes in different brain parts after treatment with sulfone-modified AuNPs were found. These studies indicate that AuNPs can be considered for the treatment of neuroinfections (Table 1) [45].

The administration time is essential when using AuNPs as potential antiviral substances. Many studies showed that AuNPs administered one hour before the infection and one hour after the infection show an antiviral effect (Table 1). In the pretreatment system, lower HHV-1 titers measured by PFU/mL were obtained compared to the treatment after the infection [28]. A study by Krzyzowska et al. showed that lactoferrin-modified 5 nm AuNPs effectively inhibited the adsorption and penetration of HHV-2 in the HaCat human keratinocyte cell line and the VK-2 vaginal epithelial cell line [28]. Furthermore, cellular protection was demonstrated by adding the maximum dose of AuNPs, which significantly inhibited viral replication [28]. Baram-Pinto et al. demonstrated the effective inhibition of HHV-1 virus replication using MES-modified AuNPs. MES-coated NPs inhibit HHV-1 entry into cells because NPs interact similarly with the HSPGs [33]. Halder et al. also demonstrated effective inhibition of HHV-1 infection in the Vero cell line using gallic acid (GA)-modified AuNPs. AuNPs-GA inhibited penetration and caused the inactivation of viral particles by preincubation with AuNPs. Furthermore, AuNPs-GA showed a similar percentage of infection inhibition compared to ACV [47].

As it is well known, HHV-1 has been linked with changes in the brain tissue similar to Alzheimer’s disease. HHV-1-induced neuroinflammation stimulates the accumulation of abnormal forms of amyloid β and results in excessive hyperphosphorylation of Tau protein. It appears that the administration of AuNPs causes a decrease in the secretion of β-secretase, which is related to the proteolytic processing of APP (amyloid precursor protein). The administration of AuNPs also decreased the formation of amyloid β-40 associated with the appearance of amyloid plaques. This makes AuNPs good therapeutic agents for the removal of neurodegenerative changes accompanying HHV-1 infection [45].

We tested the antiviral activity of tannic acid-modified AuNPs (TA-AuNPs) sized 5 and 30 nm in the Neuro-2a cell line as well as in the culture of primary neurons (Figure 3). Both cultures, when exposed to non-toxic concentrations of TA-AuNPs for 6 h before infection, showed very good inhibition of infection for the neuronal cell cultures (Figure 3), which makes TA-AuNPs very promising candidates for the treatment of HHV-1 infection. We can also conclude that while 5 nm AuNPs nanoparticles can be internalized both by active phagocyting cells within the neuronal tissue, such as microglia, and the neuronal cells themselves, the antiviral activity of 5 n AuNPs may originate from an indirect effect by influencing the viability/metabolic activity of neuronal cells. On the other hand, 30 nm AuNPs are not internalized by neuronal cells; therefore, their antiviral effects are exerted outside the cells by preventing viral infection.

## 5. Silver Nanoparticles

The antimicrobial activity of silver has been known for a long time. Compared to gold, it is less chemically stable and shows higher toxicity. Cellular toxicity depends mainly on the size and type of modification of silver nanoparticles (AgNPs). Little is known about their neurotoxicity. For many researchers, the use of AgNPs is counterintuitive due to their cytotoxicity: AgNPs < 20 nm are considered to have the highest toxicity [29,48]. This effect is contributed to the ability of small AgNPs to directly pass the cellular membranes, while larger nanoparticles (>30 nm) are actively internalized by the cells in the processes such as phagocytosis, micropinocytosis, caoevolin-dependent, and clathrin-dependent endocytosis. Studies by Gaiser and co-workers have shown that 20 nm AgNPs induce inflammation, cytotoxicity, and an increase in oxidative stress levels in a human hepatocyte cell line and in vivo in female Wistar rats [48]. Both in vitro and in vivo models treated with AgNPs have shown an increase in inflammatory cytokines such as IL-8, MIP2, IL-1RI, and TNF-α [48,49]. So far, it has been shown that AgNPs can disrupt mitochondrial function and stimulate the formation of reactive oxygen species (ROS), leading to cell apoptosis/necrosis. In addition, in vivo studies on rodents treated with AgNPs showed an increase in the expression of genes related to oxidative stress. A study by Sharma et al. 2013 showed that AgNPs with a size of 50–60 nm could cause damage to myelin [50]. It also appears that long-term exposure to 15–20 nm unmodified AgNPs does not result in increased mortality or significant toxicity in mice. In a study by Recordati et al., long-term exposure to low doses of AgNPs for 4 weeks (oral exposure) was tested. Treatment with AgNPs did not result in changes in body weight or damage to internal organs. Only blood biochemical changes such as increased urea and triglycerides occurred. In addition, lymphopenia was also observed. After a 4-week exposure to AgNPs at a dose of 1 mg/kg body weight, the highest concentrations were observed in the brain, testes, liver, and spleen [51]. The distribution of AgNPs through the body also depends on their size. The study by Park et al. showed that small nanoparticles (2 nm, 42 nm and 71 nm) when administered orally, passed into the lung, brain, liver, kidney, and testis. In contrast, large nanoparticles of 323 nm were not present in mice organs. Furthermore, increased levels of TGF-β were observed when mice were treated orally with small AgNPs [52].

In addition, the study by Dabrowska-Bouta et al., 2016, showed that exposure to 10 nm AgNPs caused changes in the expression of myelin-specific proteins, CNP, MAG, and MOG, which may be responsible for ultrastructural changes in myelin sheaths [53]. Furthermore, oxidative stress was observed in brain homogenates of rats treated with low doses of AgNPs. In vitro studies in the BBB model (primary rat brain microvascular endothelial cells, pericytes, and astrocytes) indicated a significant unsealing of the barrier and a marked decrease in the level of ZO-1 protein, which belongs to tight junctions. In astrocytes treated with AgNPs, a change in mitochondrial morphology and vacuolization was shown. In addition, it was demonstrated that in astrocytes treated with AgNPs, there was an activation of inflammation and apoptosis through modulation of the MAPK pathway or B-cell lymphoma-2 expression or mTOR activity [54].

Due to their size, AgNPs can overcome the BBB. As we know, the activation of inflammation has a protective function in response to various agents, including pathogens and toxins that have entered our bodies. In the brain tissue, inflammation, the consequence of which is the activation of the microglia and the change in the permeability of the BBB, significantly impacts the development of neurodegeneration, but also the clinical outcome of neuronal infection. Several studies have shown that inflammation can be minimized by the use of AgNPs. For example, 20–60 nm AgNPs synthesized from *Cotyledon orbiculata* aqueous extract have been shown to have anti-inflammatory effects by reducing the expression of IL-1B and IL-6 in macrophages differentiated from THP-1 cells [55]. Additionally, AgNPs with a size of 22–24 nm, obtained from a natural extract of *Viburnum opulus* L. fruits, showed anti-inflammatory and regenerative properties [56]. The anti-inflammatory effect was observed in vitro in HaCaT keratinocytes exposed to UVB, in which 25 nm silver nanoparticles synthesized with *Viburnum opulus* fruit extract modulated the release of inflammatory cytokines IL-1α and IL-6 [56]. Moreover, the authors used in vivo model of Wistar rats with a subplantar injection of carrageenan to induce the secretion of pro-inflammatory cytokines. The inflammatory effect within the plantar skin was diminished by AgNPs [56].

**Figure 4:**
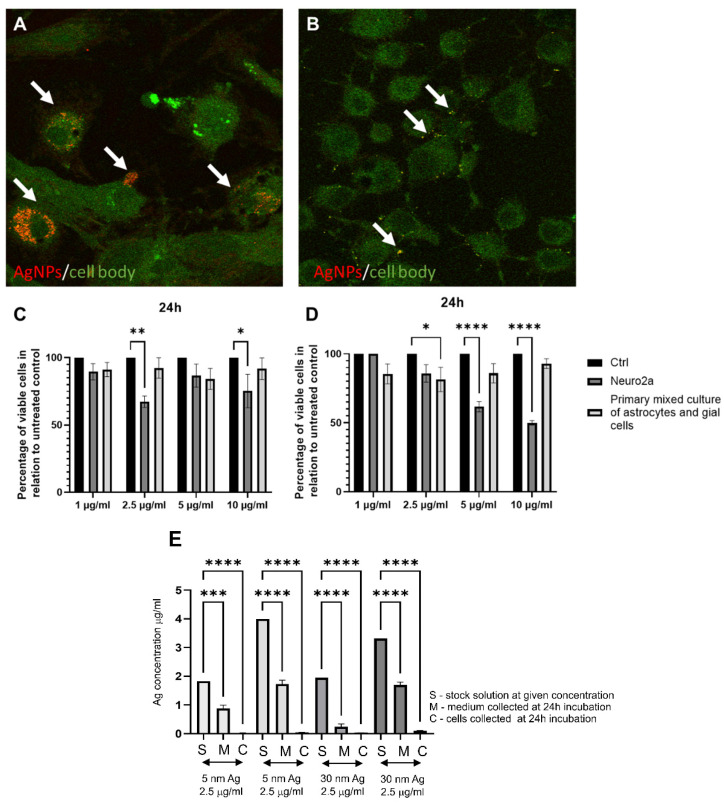
AgNPs internalization by confocal microscopy, ICP-MS and size/cell type-dependent toxicity. Primary mixed culture of astrocytes and glial cells (**A**), forskolin differentiated Neuro-2a cells (**B**) were treated with AgNPs with Tannic Acid (TA) 30 nm at a concentration of 2.5 µg/mL for 24 h. For microscopic detection of silver NPs, cells were plated on slides for 18 h before exposure to NPs. After 24 h of exposure to 1 µg/mL of TA-AgNPs, the medium was discarded, and the cells were fixed with acetone:methanol; 1:1 (Sigma-Aldrich, St. Luis, MO, USA). The images were captured on an upright Leica SP8 resonant scanning confocal system (Leica Microsystem, Wetzlar, Germany). The pinhole was set to 1 AU. Nanoparticles were visualized in a reflection mode (red color) using a 638 nm laser line with a 40× oil immersion objective (NA 1.30), while cellular autofluorescence (green color) was used to visualize the cell shape. Arrows point to nanoparticles (red). The acquisition was performed in a sequential mode. The AgNPs-TA 5 nm (**C**) and 30 nm (**D**) cytotoxicity was estimated with the MTT test. The cell viability was measured at Neuro-2a and primary mixed culture of astrocytes and glial cells incubation with various concentrations of AgNPs-Ta for 24 h. Control is a non-treated cell culture. (**E**) Inductively coupled plasma mass spectrometry (ICP-MS) for Ag content in stock solutions, media, and cells collected at 24 h post incubation. Data from three independent experiments are presented as mean ± SEM. Two way ANOVA test *p* ≤ 0.05 *, *p* ≤ 0.01 **, and extremally significant at *p* ≤ 0.001 *** or *p* ≤ 0.0001 ****. The figure presents our own unpublished data.

We performed studies with mixed cultures of astrocytes, glial cells, and Neuro2a cell lines to understand how 30 nm AgNPs can influence neuronal and glial cell viability and to check if they can be internalized by the cells (Figure 4). In comparison to TA-AuNPs of the same 30 nm size, both glial and neuronal cells internalized AgNPs more efficiently (Figure 4A,B,E). On the other hand, the internalization of 5 nm TA-AgNPs was less efficient in comparison to 5 nm TA-AuNPs (Figure 2E and Figure 4E). However, taking into account differences in sizes (5 nm vs. 30 nm), small AgNPs were internalized more efficiently than larger sizes.

A number of studies indicate that AgNPs have successfully penetrated the BBB, but their toxicity often limits their use. However, the studies by Gonzales-Carter et al. showed that citrate-modified AgNPs are internalized and dissolved by microglia. Furthermore, silver ions are detoxified to form Ag_2_S (silver sulfide), deposited on the surface of NPs. Interestingly, in microglial cells treated with AgNPs and LPS, there was a decrease in the production of ROS, nitric oxide, and TNFα. These studies suggest that thanks to the known detoxification mechanism, it may be possible to use AgNPs to treat neurodegenerative diseases, where microglia play a major role [57].

Functionalization of AgNPs with plant-derived polyphenols can significantly reduce their toxicity. An example of such a compound is tannic acid (TA). It is a plant polyphenol whose monomer is gallic acid. Its neuroprotective effect has been demonstrated. In rats with experimentally induced sporadic dementia of Alzheimer’s type (SDAT), TA was administered and improved cognitive abilities. Moreover, rats treated with TA showed a lower percentage of neuronal degeneration and reduced levels of pro-inflammatory cytokines (Il-12 and Il-4). In addition, TA administration increased the expression levels of Akt and pAkt, protecting neurons from death [58]. Similar results were obtained on transgenic mice with an Alzheimer’s disease model. Administration of TA for half a year resulted in reduced behavioral disturbances and maintained recognition of new objects. Interestingly, the behavioral changes were also reflected in changes in amyloid β. In addition, a decrease in secretase activity and microgliosis was observed. The results of these studies confirm the validity of the use of TA in the treatment of Alzheimer’s disease, reducing symptoms and neuroinflammation [59]. The study by Szymanska et al. also showed effective inhibition of HHV-1 and HHV-2 infection using 33 nm AgNPs modified with TA. The NPs were suspended in a hydrogel. The percentage of infection inhibition was checked in the HaCat human keratinocyte cell line. Inhibition of replication, cell-to-cell transmission, and viral entry was proven. We also tested antiviral activity of tannic acid-modified AgNPs sized 5 and 30 nm in the Neuro-2a cell line as well as in the culture of primary neurons (Figure 3). Neuro-2a cell line, when exposed to a non-toxic concentration of TA-AgNPs for 6 h before infection, showed very good inhibition of infection for the neuronal cell cultures (Figure 3A), while primary neurons were not protected from HHV-1 infection only by 5 nm TA-AgNPs (Figure 3B), which may be related with higher toxicity of AgNPs 5 nm in comparison to 5 nm AuNPs (Figure 2 and Figure 4). Taking into account the internalization of both 5 and 30 nm TA-AgNPs by neuronal and glial cells, we can conclude that the antiviral effect of TA-AgNPs is exerted both directly (preventing infection) and indirectly due to toxicity (5 nm AgNPs) or by influencing the viability/metabolic activity of neuronal cells (30 nm TA-AgNPs).

In addition, an in vivo herpes genital model in mice was also used, where positive antiviral results were also obtained [60]. Orlowski et al. demonstrated the anti-HHV-2 activity of tannic acid-modified AgNPs (TA-AgNPs) both in vitro and in vivo, the latter using models of genital HSV-2 infection [61,62]. The mechanisms of antiviral action of TA-AgNPs included blocking of virus attachment, entry, as well as induction of antiviral cytokine and chemokine production (Table 1). Additionally, TA-AgNPs turned out to be good activators of the immune response since they were also able to overcome the inhibition of DC maturation by live or inactivated HHV-2, normally observed in this model of infection [29]. Therefore, we demonstrated that tannic acid-modified AgNPs might act not only as an effective microbicide but also as a novel class of nano-adjuvants.

## 6. Conclusions

Neuroinfections of herpesvirus etiology are a fundamental problem for modern medicine, mainly due to their association with neurodegenerative diseases. Available therapies based on nucleotide analogs may be insufficient due to other comorbidities related to herpesvirus infections (such as encephalitis) and a lack of efficient vaccination strategies. Using different substances to modify the surface of nanoparticles or by steering their sizes, we can influence their ability to cross BBB and to become internalized by different neuronal cell types. Since herpesvirus infection, both primary and latent, involves the neuronal cells present in the skin, we should keep in mind the possibility of influencing the outcome of HHV-1/HHV-2 local infection by topical use of modified Au/AgNPs. Further studies are required for good refinement as well as more in vivo studies on the bioaccumulation, biodistribution, and cytotoxicity of noble metal NPs.

## Figures and Tables

**Table 1:** Antiviral mechanism by functionalized gold or silver nanoparticles.

Functional Group/Modification/Metal	Potential Antiviral Mechanism	Sources
MES (Au) ^1^	inhibits viral attachment and cell-to-cell spreading	[33]
LT (Au/Ag) ^2^	inhibits viral attachment and entry into the host cell	[28]
MUS (Au) ^3^	mimics the heparan sulfate proteoglycans (HSPG) binding site on the cell surface	[34]
MUS-Ot(Au) ^4^	prevents the virus from binding and/or entering the cell	[35]
Amin (Au) ^5^	blocks receptor-binding domain	[36]
SiO_2_ (Au) ^6^	blocks penetration of the virus into the cell due to a change in the membrane potential.	[37]
TA (Ag) ^7^	blocks virus attachment, entry, and cell-to-cell spreading	[29]
CD(Au) ^8^	prevents the virus from binding and/or entering the cell	[35]

^1^ MES-3-mercapto-ethyl sulfonate, ^2^ MUS-mercapto-undecansulfonic acid, ^3^ LT-lactoferrin, ^4^ MUS-Ot-Octanethiol, ^5^ Amin-mercaptooctan-1-aminium, ^6^ SiO_2_-silicon dioxide, ^7^ TA-tannic acid, ^8^ CD-β-cyclodextrins.
